# Fine Mapping of *qd1*, a Dominant Gene that Regulates Stem Elongation in Bread Wheat

**DOI:** 10.3389/fgene.2021.793572

**Published:** 2021-11-29

**Authors:** Yongdun Xie, Weiwei Zeng, Chaojie Wang, Daxing Xu, Huijun Guo, Hongchun Xiong, Hanshun Fang, Linshu Zhao, Jiayu Gu, Shirong Zhao, Yuping Ding, Luxiang Liu

**Affiliations:** National Engineering Laboratory for Crop Molecular Breeding, Institute of Crop Sciences, Chinese Academy of Agricultural Sciences, National Center of Space Mutagenesis for Crop Improvement, Beijing, China

**Keywords:** stem elongation, *qd1* gene, fine mapping, molecular markers, wheat

## Abstract

Stem elongation is a critical phase for yield determination and, as a major trait, is targeted for manipulation for improvement in bread wheat (*Triticum aestivum* L.). In a previous study, we characterized a mutant showing rapid stem elongation but with no effect on plant height at maturity. The present study aimed to finely map the underlying mutated gene, *qd1*, in this mutant. By analyzing an F_2_ segregating population consisting of 606 individuals, we found that the *qd1* gene behaved in a dominant manner. Moreover, by using the bulked segregant RNA sequencing (BSR-seq)-based linkage analysis method, we initially mapped the *qd1* gene to a 13.55 Mb region on chromosome 4B (from 15.41 to 28.96 Mb). This result was further confirmed in F_2_ and BC_3_F_2_ segregating populations. Furthermore, by using transcriptome sequencing data, we developed 14 Kompetitive Allele-Specific PCR (KASP) markers and then mapped the *qd1* gene to a smaller and more precise 5.08 Mb interval from 26.80 to 31.88 Mb. To develop additional markers to finely map the *qd1* gene, a total of 4,481 single-nucleotide polymorphisms (SNPs) within the 5.08 Mb interval were screened, and 25 KASP markers were developed based on 10x-depth genome resequencing data from both wild-type (WT) and mutant plants. The *qd1* gene was finally mapped to a 1.33 Mb interval from 28.86 to 30.19 Mb on chromosome 4B. Four candidate genes were identified in this region. Among them, the expression pattern of only *TraesCS4B02G042300* in the stems was concurrent with the stem development of the mutant and WT. The *qd1* gene could be used in conjunction with molecular markers to manipulate stem development in the future.

## Introduction

Facing a growing population and changing climate, we must increase crop yields to secure the food supply for the future. According to the FAO, the world will need to feed 9.1 billion people by 2050 (Food and Agriculture Organization of the United Nations, 2021), and crop production needs to double to meet the projected demands of the increasing population, dietary shifts, and increasing consumption of biofuels (Ray et al., 2013). Currently grown worldwide, wheat has become the most important food source since it is a major source of carbohydrates in the majority of countries and is ideal for processing into different food products (Igrejas et al., 2020). Although global wheat production has risen over two and a half times since 1960, increasing the wheat yield potential remains a major goal for breeders and geneticists (Reynolds et al., 2009).

Stem development is critical for final yield production. Wheat stems provide physical support for spikes and lodging resistance. Macronutrient and micronutrient transport from the soil to the aboveground portions of plants takes place in the stems (Singh et al., 2020), and stem reserves from plant assimilation preanthesis are increasingly recognized as important sources of carbon for grain filling, especially in stressful environments (Blum, 1998). Due to the importance of wheat stem development, this process has been recognized as an important target for improvement.

To further characterize the dynamic changes in wheat growth, the elongation phase, from the terminal spikelet stage to anthesis, is divided into seven stages (Guo et al., 2018). By using 210 wheat accessions, researchers qualified 75 plant growth-associated traits that can be manipulated to alter grain yields at these seven stages of the elongation phase (Guo et al., 2018). Moreover, wheat stem elongation is affected by a series of environmental factors (Jamieson et al., 1998; Whitechurch et al., 2007). Temperature is one of the most important factors affecting stem elongation (Shimizu and Hisamatsu, 2009). The temperature response of wheat is highly heritable and affects both the final height and the timing of stem elongation (Kronenberg et al., 2021). In addition, vernalization and photoperiod have combined effects on stem elongation. Compared with the photoperiod, the vernalization response has a stronger effect on the duration of the vegetative phase, while photoperiod sensitivity can prolong the stem elongation phase by up to 23% (Steinfort et al., 2017). Unlike many other species, low red/far-red ratios delay wheat stem growth, especially peduncle elongation (Ugarte et al., 2010). Photosynthetic traits have also been shown to have weaker associations with plant stem length in wheat (Yu et al., 2020).

A series of wheat genes has been shown to regulate stem growth, and many of these genes reduce plant height due to the importance of the shortened plant height contribution to lodging resistance. To date, more than 20 wheat reduced height (*Rht*) genes have been identified (Mo et al., 2018), and *Rht-1*, *Rht-2*, and *Rht-8* are the three most important semidwarfing genes according to their distribution in widely adapted varieties (Tang et al., 2009). By using a whole-genome association mapping method, a variety of plant height-associated loci were identified (Zanke et al., 2014). In addition to plant height-associated genes, *VRN1* (Harris et al., 2017), *PPD1* (Harris et al., 2017), *TB1* (Dixon et al., 2020), cinnamoyl-CoA reductase-encoding genes (Ma, 2007), and *TaCOLD1* (Dong et al., 2019) have also been found to function in regulating wheat stem growth. Additionally, all of these genes that regulate stem growth affect the final plant height of mature plants.

We screened and characterized a rapidly developing wheat mutant, *qd*, which showed an accelerated growth rate but had a final plant height equivalent to that of the wild-type (WT) control (Zhang et al., 2016). Transcriptome analysis revealed that benzoxazinoids have a potential role in regulating stem development in *qd* (Xu et al., 2021). In this study, we finely mapped the *qd1* mutation responsible for regulating this phenotype. Candidate genes can be used in breeding programs to manipulate stem growth in the future.

## Materials and Methods

### Plant Materials and Phenotypic Observations

The *qd* mutant was developed through ion beam mutagenesis as described in our previous report (Zhang et al., 2016). The *qd* mutant and WT Lunxuan 987 were used to construct a mapping population. All plants used in this study were grown at the experimental station of the Institute of Crop Sciences, Chinese Academy of Agricultural Sciences (CAAS), Beijing, and studied in 2017, 2018, and 2019. Twenty seeds of all genotypes in the mapping population were planted in 2 m rows, with 25 cm spacing between rows. Every 20 rows contained one row of the WT and one row of the *qd* mutant, which were used for phenotypic control. The phenotypes of the plants composing the mapping population were characterized by measuring the plant height at both the booting stage and the mature stage. The fresh weight and dry weight of the roots, stems, and leaves of five individual *qd* and WT plants were measured at the jointing stage, booting stage, heading stage, and filling stage.

### Bulked Segregant RNA Sequencing (BSR-Seq) Analysis

At the booting stage, 20 plants representing typical WT phenotypes and 20 plants representing typical *qd* phenotypes were sampled in the field and immediately placed in liquid nitrogen. At the same time, 15 WT and 15 *qd* plants were pooled in the same way. Total RNA was isolated from the bulked samples using an RNeasy^®^ Plant Mini Kit (Qiagen, Germany). The purification of RNA samples and construction of sequencing libraries were performed as described previously. RNA sequencing was carried out on an Illumina HiSeq X series platform. The clean reads were subsequently aligned to the wheat reference genome (IWGSC RefSeq v1.0, https://wheat-urgi.versailles.inra.fr/Seq-Repository/Assemblies) by using STAR v2.3.0e software (Dobin et al., 2013). Single-nucleotide polymorphisms (SNPs) were subsequently called by using GATK v3.1-1 software (Mckenna et al., 2010). The original transcriptome data have been uploaded to NCBI.

### DNA Isolation and Genome Resequencing

The leaves of 30 WT and *qd* plants were collected and pooled together. DNA was isolated using the cetyl-trimethylammonium bromide (CTAB) method. Two replicate biological samples of the WT and mutant were sent for genome resequencing at Annoroad Gene Technology in Beijing. Sequencing was performed on an Illumina HiSeq 2000 platform at a 10x depth. The original genome resequencing data have been uploaded to NCBI.

### Identification of SNPs Between the WT and *qd*

High-quality clean reads were used for SNP identification between the WT and *qd*. SNPs were manually screened from the reads sequenced more than 10 times via IGV visual software (Guan and Sung, 2016). The chromosomal positions and nucleotide variations of each SNP between the initial positioning intervals were recorded in Excel 2010 software.

### Development of Kompetitive Allele-Specific PCR (KASP) Markers and Genotyping

There were two criterial rules for the SNPs selection from both transcriptome data and the genome resequencing data. The first one was that the genotypes of WT and *qd* can be finely differentiated by KASP makers. The second rule was that the mapping population can be clearly differentiated by KASP makers. Genomic sequences (200 bp) flanking the targeted SNPs were used as templates for designing PCR primers. The primers were designed with the online tool PolyMarker (http://www.polymarker.info/). For each SNP site, the reverse primer was set to “common”. Forward primer A ended with the variant nucleotides for the WT genotype, while forward primer B ended with the variant nucleotides for the *qd* genotype. The FAM sequence (5′-GAA​GGT​GAC​CAA​GTT​CAT​GCT-3′) was added to the 5′ end of forward primer A, and the HEX sequence (5′-GAA​GGT​CGG​AGT​CAA​CGG​ATT-3′) was added to the 5′ end of forward primer B. All primers were synthesized by Sangon Biotech.

PCR was performed in a 384-well system. Each reaction consisted of 2.5 µL of 2x KASP Master Mix (LGC Genomics, Middlesex, UK), 0.06 µL of KASP Primer Mix (forward primer A (100 μM):forward primer B (100 μM):reverse primer R (100 μM):ddH2O = 12:12:30:46), 0.04 µL of 25 mM MgSO4, and 2.4 µL of DNA template. The cycling program was set to 95°C for 15 min, 10 cycles of 95°C for 20 s followed by 61°C for 30 s, and 30 cycles of 95°C for 10 s followed by 57°C for 1 min. After the reactions, the fluorescence signals were scanned via FLUOstar Omega SNP (BMG Labtech), and genotype identification was performed by Omega software (v.5.11 R3).

### Linkage Map Construction

The QTL IciMapping v4.2 program (http://www.isbreeding.net/) was used for genetic map construction. The BIP (QTL mapping in biparental populations) function was used in this study, with the default settings used for all other parameters. The logarithm of odds (LOD) score was set to a threshold of 2.5, and the Kosambi map function was chosen to calculate the mapping distance from recombination frequencies.

### Candidate Gene Transcriptional Analysis With Quantitative Real-Time RT-PCR (qPCR)

RNA was isolated following the protocol described in the BSR-seq analysis section. cDNA was synthesized using TransScript^®^ All-in-One First-Strand cDNA Synthesis SuperMix for qPCR (one-step gDNA removal) (TransGen Biotech, China) according to the manufacturer’s instructions. cDNA synthesis was conducted in a 20 µL volume composed of 4 µL of 5x TransScript® All-in-One Super Mix for qPCR, 1 µL of gDNA Remover, 2 µL of RNA template, and 13 µL of RNase-free water. The cDNA synthesis program was as follows: 42°C incubation for 15 min followed by 85°C incubation for 15 s. The cDNA products were standardized to 200 ng μl^−1^ until further use. The primers used for qPCR analysis of candidate genes are listed in Supplementary Table S1. A CFX 96 Real-Time System (Bio-Rad, USA) and TransScript^®^ Top Green qPCR SuperMix (TransGen Biotech, China) were used to perform qPCR and analyze the generated data. The *actin* gene was used as an internal control. We included at least three technical replicates for each RNA sample per plate. The comparative threshold 2^−ΔΔCT^ method was applied to quantify relative gene expression.

## Results

### Stem Elongation Was Quicker in the *qd* Mutant Than in the WT

**FIGURE 1:**
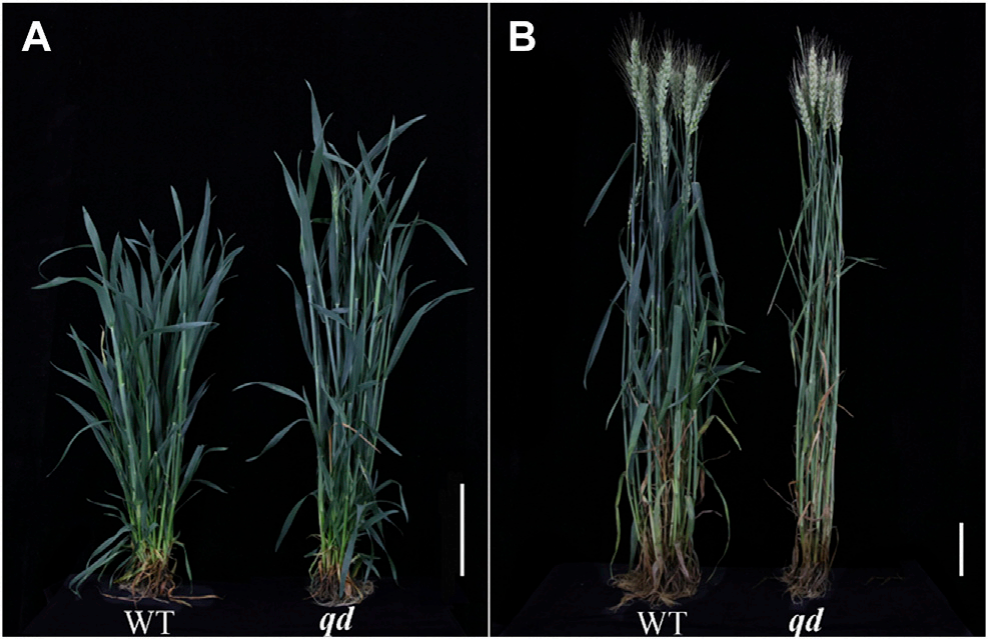
Heights of WT and *qd* plants at various stages. Bar = 8 cm. **(A)** Typical plants at the booting stage; **(B)** Typical plants at the mature stage.

**FIGURE 2:**
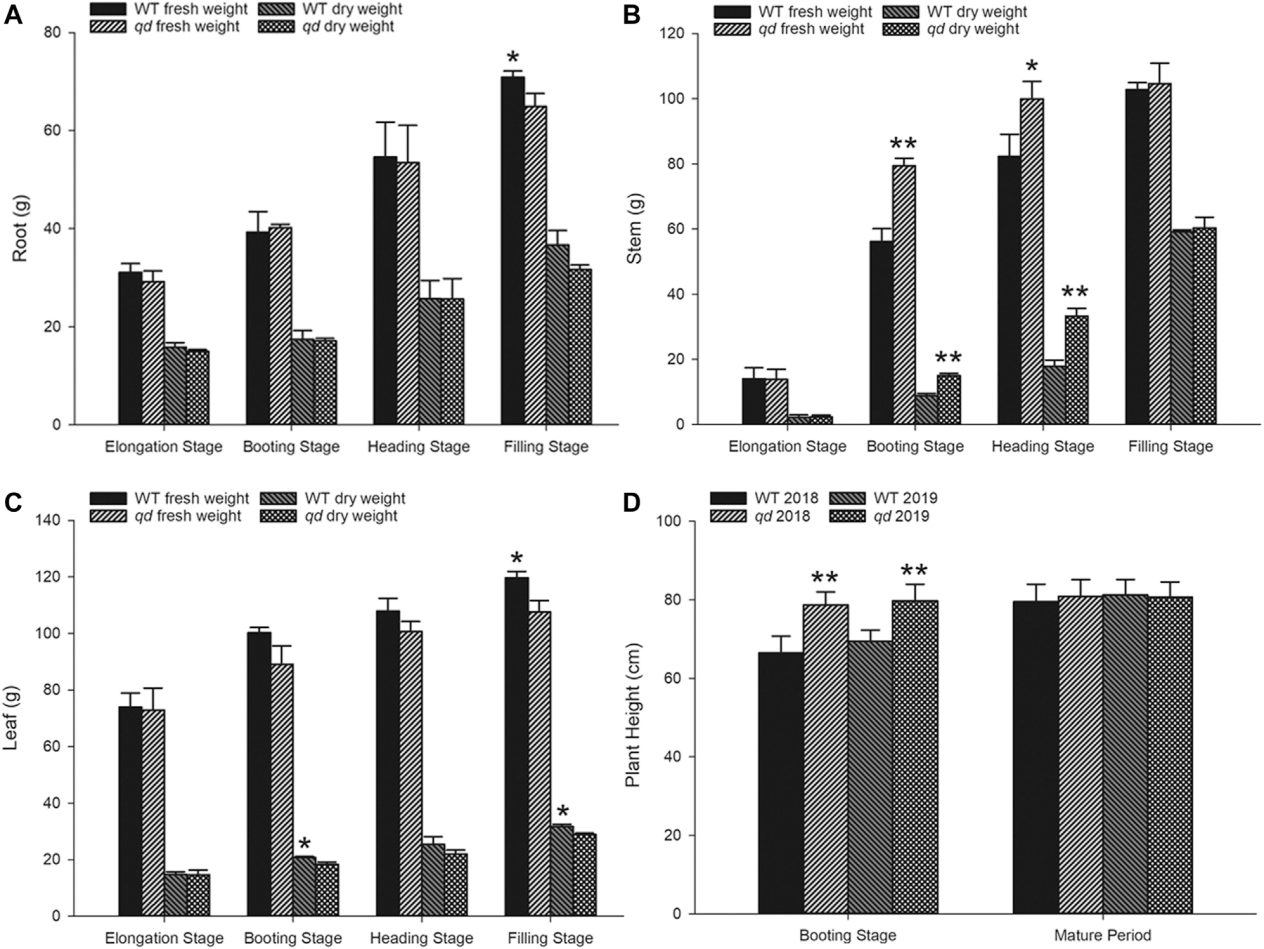
Phenotypic data of WT and *qd* plants at various stages. **(A)** Root weight comparison between *qd* and the WT; **(B)** Stem weight comparison between *qd* and the WT; **(C)** Leaf weight comparison between *qd* and the WT; **(D)** Plant height comparison between *qd* and the WT Note: “**” indicates that the difference between *qd* and the WT is significant at the 0.01 level; “*” indicates that the difference between *qd* and the WT is significant at the 0.05 level.

As we reported previously (Zhang et al., 2016), the *qd* plants were taller than the WT plants from the booting to heading stages but were similar in height to the WT at maturity, as shown in Figure 1 and Figure 2D.

Neither the fresh weight nor the dry weight of the roots differed between *qd* and the WT before the heading stage, but compared with that of *qd* roots, the fresh mass of the WT roots was greater at the filling stage, as shown in Figure 2A. Similar to the plant height phenotype, both the fresh weight and dry weight of the stems of *qd* were significantly higher than those of the WT at the booting stage and heading stage, while there was no difference at the filling stage, as shown in Figure 2B. However, the dry leaf weight of the WT was higher than that of *qd* at the booting and filling stages, and the fresh leaf weight of the WT was also higher than that of *qd* at the filling stage, as shown in Figure 2C.

### *qd1* Is a Phenotypically Dominant Gene

**FIGURE 3:**
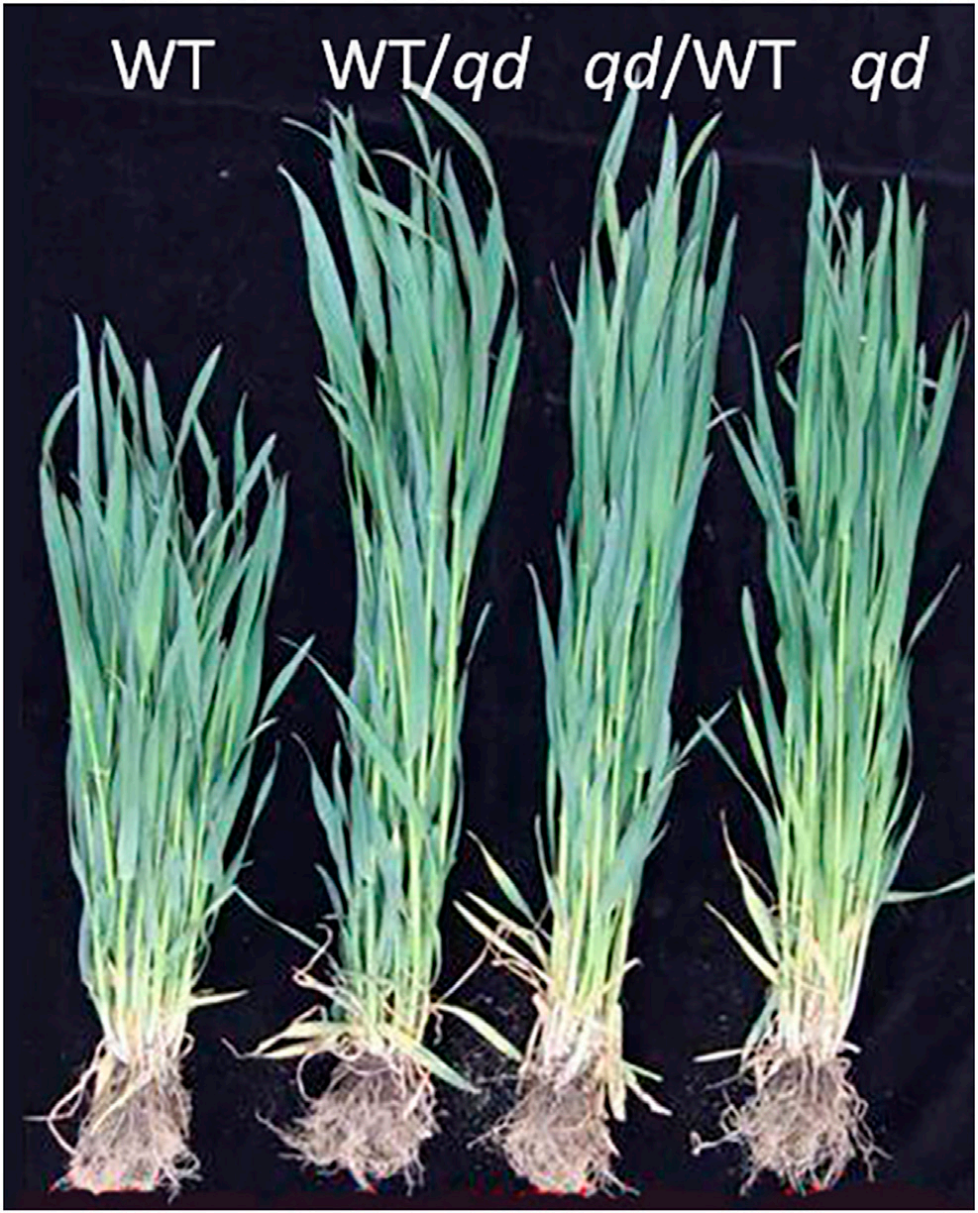
Plant heights of WT, *qd*, and F_1_ plants at the booting stage.

As shown in Figure 3, the phenotype of the F_1_ plants derived from a cross of the WT and *qd*, regardless of whether *qd* served as the male or female parent, was the same as that that of *qd*. This result suggested that the phenotype of *qd* was inherited in a dominant manner. A total of 606 F_2_ individuals from a WT x *qd* cross were characterized according to their phenotypes. The results showed that the phenotype of 454 plants was the same as that of *qd*, and the phenotype of 152 plants was the same as that of the WT. These results fit a 3:1 segregation model with a χ^2^ value of 0.0022 and a *p* value of 0.9626, which means that the stem development-regulating gene *qd1* is a phenotypically dominant gene.

### BSR-Seq-Based Linkage Analysis

**FIGURE 4:**
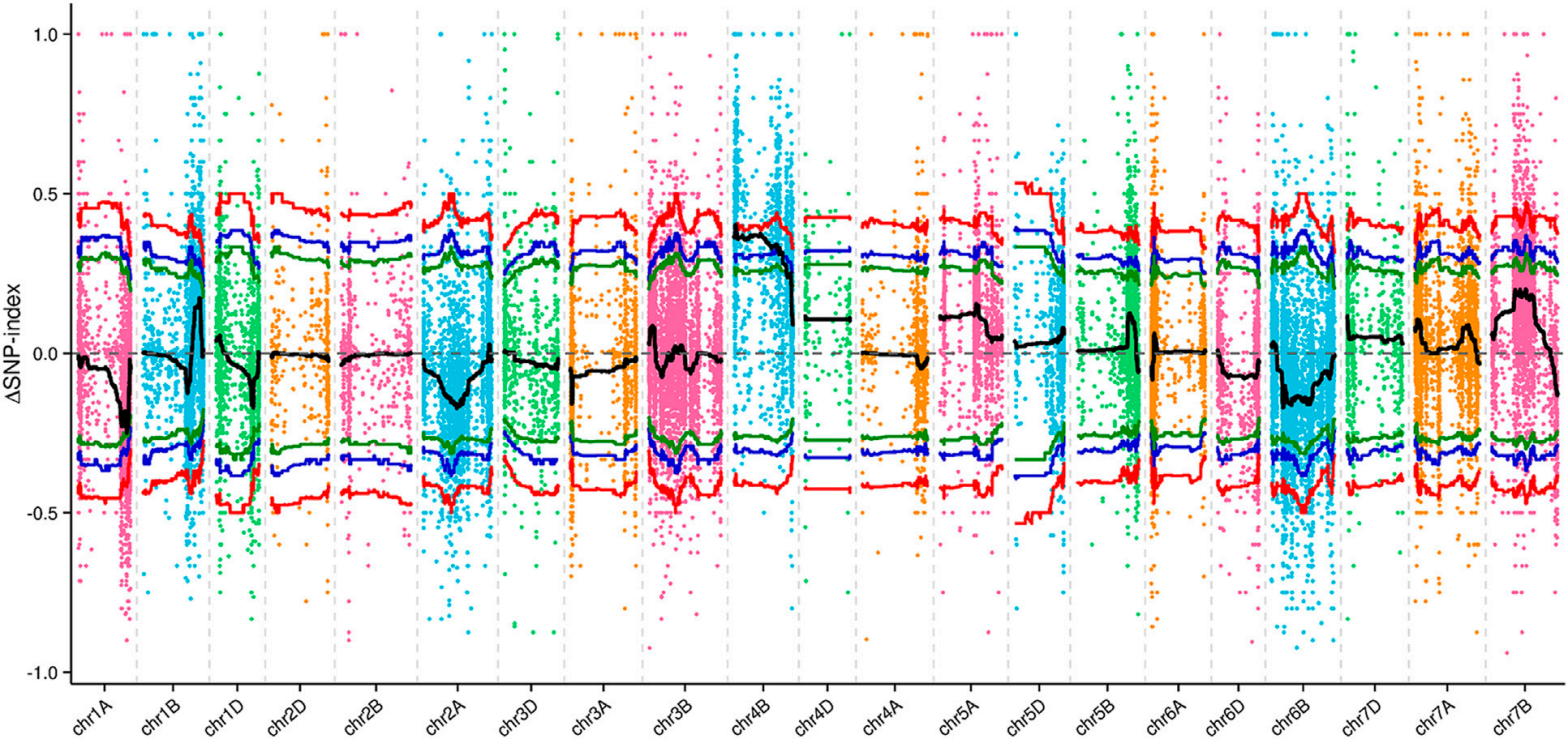
ΔSNP index values of all SNPs across the chromosomes. Note: The colored dots represent the SNP index values for this region. The black line represents the fitted SNP index value. The green, blue, and red lines represent the threshold lines with confidence levels of 0.90, 0.95, and 0.99, respectively.

From the F_2_ population, 20 plants with a *qd*-type phenotype and 20 plants with a WT phenotype were collected and formed into 2 pools. At the same time, 15 *qd* and WT plants were pooled together. The RNA of these four pools was sequenced, and 32899 HC SNPs were screened and used for linkage analysis. By using the QTL-seq analysis method, we mapped the *qd1* locus to a 13.55 Mb region on chromosome 4B, from 15.41 to 28.96 Mb, with the confidence level set to 0.99, as shown in Figure 4.

**FIGURE 5:**
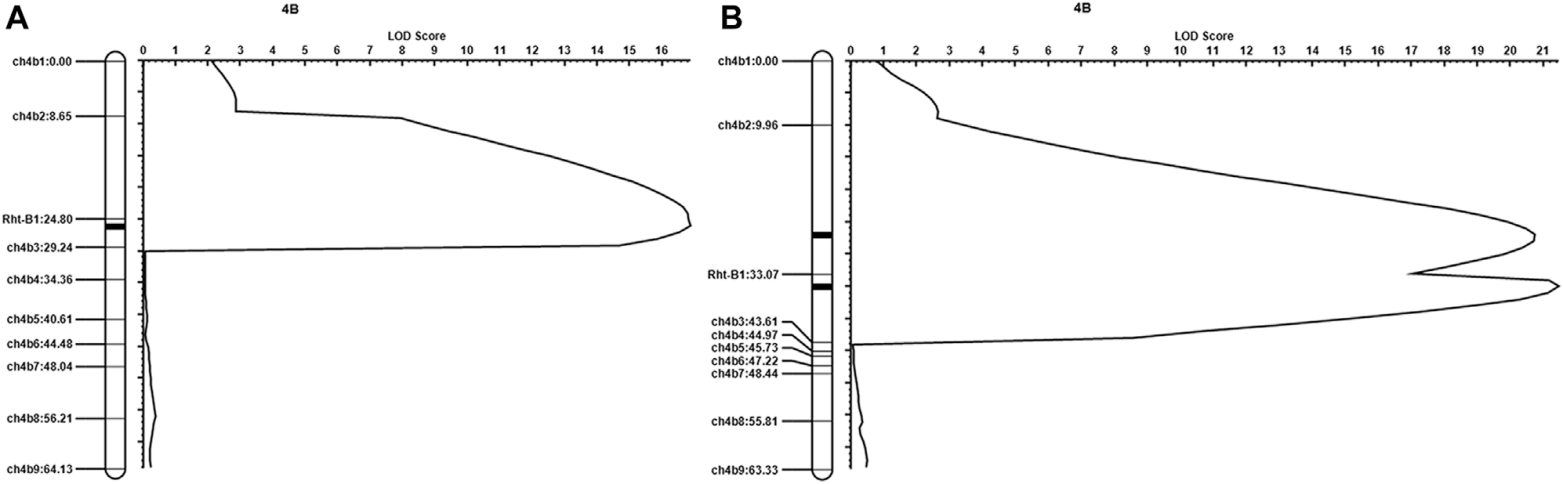
QTL mapping results **(A)** Results from the WT/*qd* F_2_ population; **(B)** results from the BC_3_F_2_ (*qd*/WT//WT///WT////WT) population.

To confirm the above results, we designed nine KASP markers distributed evenly across chromosome 4B based on transcriptome sequencing data, which are shown in Supplementary Table S2 (markers ch4b1∼9). As we reported previously (Zhang et al., 2016), the genotype of the *Rht-B1* gene was different between *qd* and WT. We also used the Rht-B1 marker (the sequence of which is listed in Supplementary Table S2) for this analysis. With these 10 markers, we subjected 352 individuals from the F_2_ population (WT/*qd*) to QTL analysis. As shown in Figure 5A, a QTL with a LOD value of 16.87 between ch4b2 and ch4b3 was detected. The phenotypic variance explained (PVE) by this QTL in the F_2_ population was 18.60%. When 361 individuals from the BC_3_F_2_ (qd/WT//WT///WT////WT) population were used for QTL analysis, we detected two QTLs. The first QTL was between ch4b2 and Rht-B1, with a LOD value of 20.73, and the second QTL was between Rht-B1 and ch4b3, with a LOD value of 21.46, as shown in Figure 5B. The PVEs of these two QTLs were 15.16 and 10.91%, respectively. These results suggested that there was a QTL between ch4b2 and ch4b3, with a physical position within 18.01–39.02 Mb on chromosome 4B. This localization interval overlapped with the analogous results of our transcriptome association analysis. Therefore, we took the position from 15.41 to 39.02 Mb as the initial positioning interval for the *qd1* locus.

### Localization of the *qd1* Locus Within the Initial Positioning Interval

**FIGURE 6:**
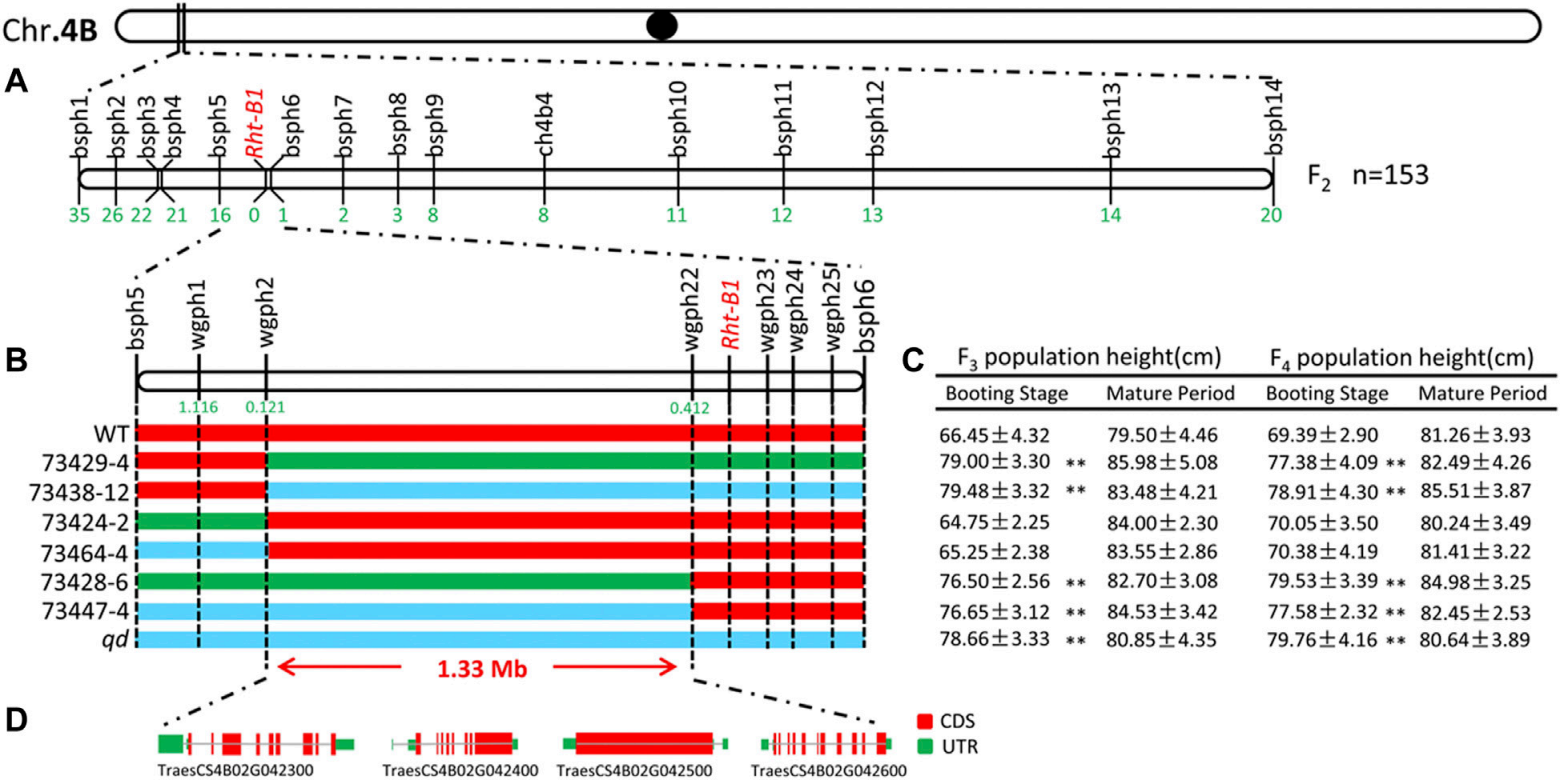
Fine mapping of the *qd1* gene. Note: **(A)** Validation of the *qd1* target interval via the F_2_ population. The numbers below the vertical lines represent the numbers of recessive single-strand recombinants in the F_2_ population. **(B)** Genotyping of the F_3_ and F_4_ populations. Red indicates the WT genotype, green indicates the heterozygous genotype, blue indicates the *qd* genotype, and the numbers below the vertical lines represents the recombination frequencies. **(C)** Phenotypic data of the plants composing the F_3_ and F_4_ populations. “**” indicates differences between the populations that are significant at the 0.01 level. **(D)** Gene annotation within the localization interval.

From the transcriptome sequencing data, we screened 121 SNPs within the initial positioning interval. Fourteen KASP marks were successfully developed and named bsph1 ∼14, as listed in Supplementary Table S2. Fifty-nine recombinant individuals from the F_2_ population were detected. Three recombinant individuals at the markers bsph6, bsph7 and bsph8 were detected. This result indicated that the *qd1* locus was located in a 5.08 Mb interval, from 26.80 to 31.88 Mb on chromosome 4B, as shown in Figure 6A.

### Development of KASP Markers Based on Genomic Resequencing Data

We pooled the DNA samples from 30 *qd* plants and 30 WT plants and subjected the pooled sample to genome resequencing. Two biological repeats were included for both *qd* and the WT. By using genomic resequencing data, we screened 4,481 SNPs within the 5.08 Mb interval (from 26.80 to 31.88 Mb). Among these SNPs, 25 KASP markers (as listed in Supplementary Table S2, namely, wgph1∼27) were developed and used for further fine mapping analysis.

### Fine Mapping of the *qd1* Locus

In the year of 2018 and 2019, the F_3_ (2,338 individuals) and F_4_ (4,123 individuals) populations were analyzed with the newly developed KASP markers. In total, 182 recombinant individuals from six recombinant lines were identified. The plant heights of recombinant lines displaying heterozygous or *qd* genotypes between markers wgph2 and wgph22 significantly differed from that of the WT plants at the booting stage, as shown in Figures 6B,C. The recombination rates of wgph2 and wgph22 were 0.121 and 0.412, respectively. Therefore, the *qd1* locus was mapped to within a 1.33 Mb interval from 28.86 to 30.19 Mb on chromosome 4B.

### Candidate Genes for the *qd1* Locus

**FIGURE 7:**
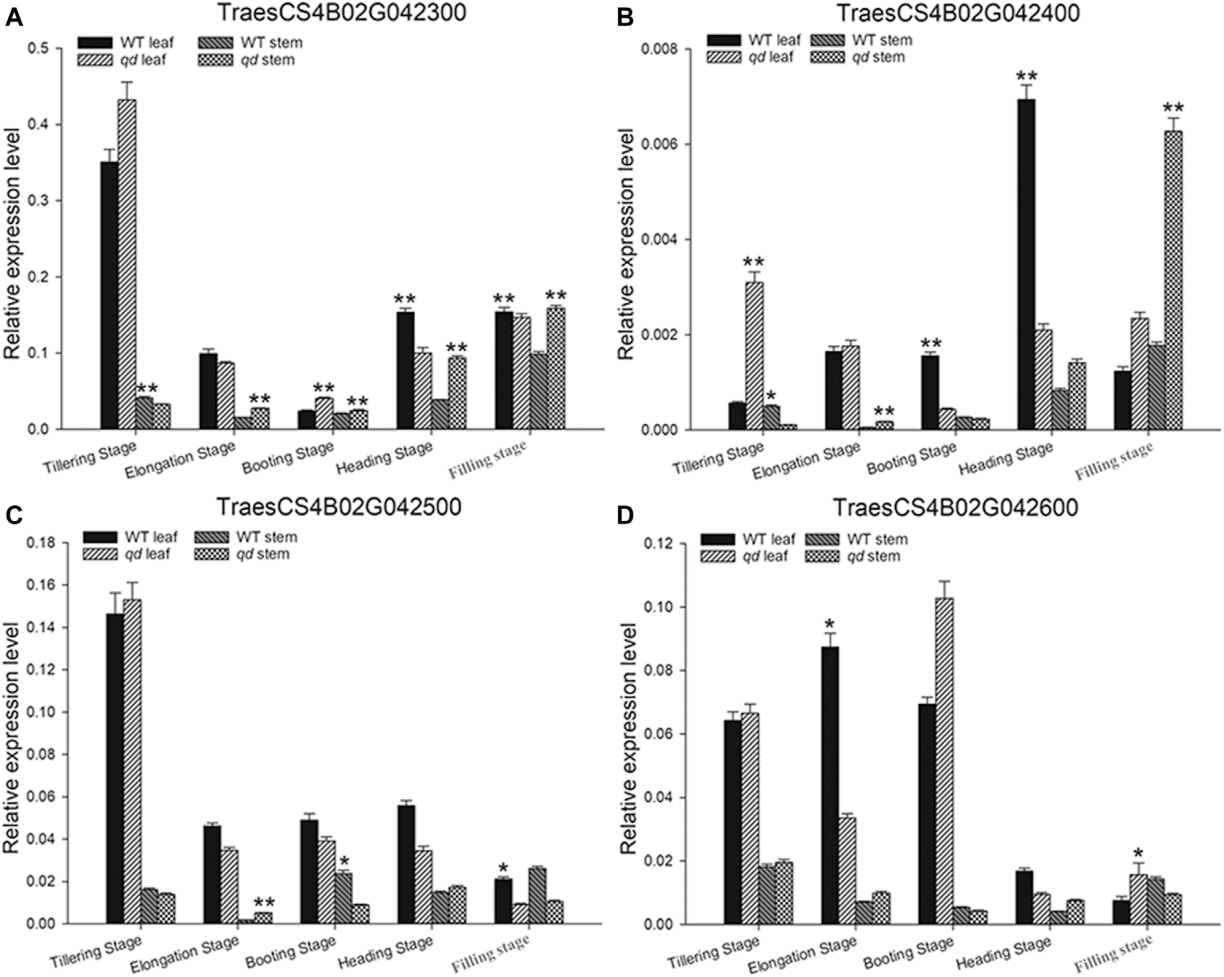
Expression analysis of candidate genes in different developmental stages and tissues. **(A)**–**(D)** indicate the genes *TraesCS4B02G042300*, *TraesCS4B02G042400*, *TraesCS4B02G042500*, and *TraesCS4B02G042600*, respectively. “*” indicates that the differences between *qd* and the WT are significant at the 0.05 level; “**” indicates that the differences between *qd* and the WT are significant at the 0.01 level.

In the targeted 1.33 Mb region on chromosome 4B, we identified four genes, namely, TraesCS4B02G042300, TraesCS4B02G042400, TraesCS4B02G042500 and TraesCS4B02G042600, as listed in Table 1 and shown in Figure 6D. We further analyzed the expression levels of these four genes in both the leaves and stems. As shown in Figure 7, only the expression pattern of TraesCS4B02G042300 in the stems was concurrent with the stem development of *qd* and the WT.

**TABLE 1:** Information about the basic candidate genes.

Gene ID	Start	End	Length(bp)	Subcellular localization	Readable-Description
TraesCS4B02G042300	28949360	28960436	11077	Chloroplast	Oxysterol-binding protein, putative
TraesCS4B02G042400	29264105	29270952	6,848	Cytoplasm, Chloroplast	Phosphatidylinositol-4-phosphate 5-kinase, putative
TraesCS4B02G042500	29673022	29674968	1947	Chloroplast, Nucleus	Fantastic four-like protein
TraesCS4B02G042600	29709859	29715180	5,322	Cytoplasm, Chloroplast	RuvB-like helicase

**FIGURE 8:**
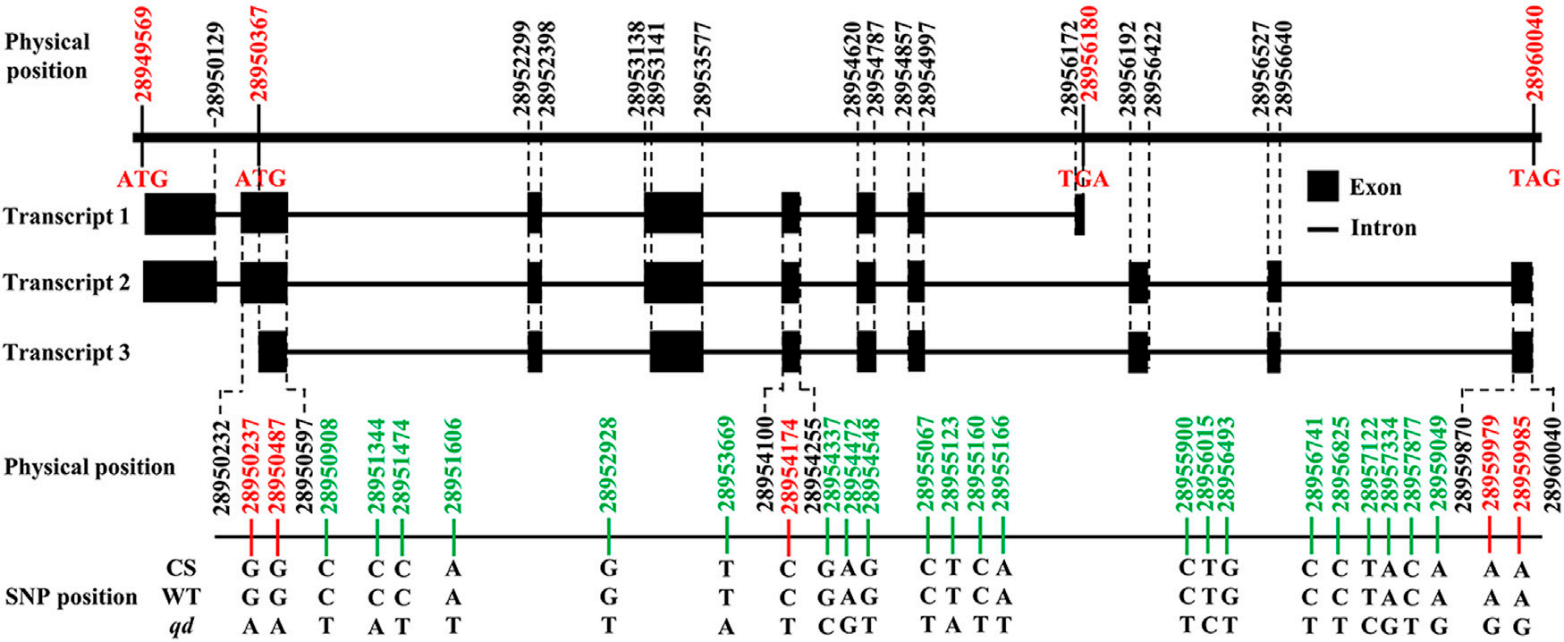
Gene structure of TraesCS4B02G042300.

According to the gene annotation results, there were 3 transcripts of this gene, as shown in Figure 8. We detected five SNPs in the exon regions of TraesCS4B02G042300. However, the functions of these SNPs need to be further explored.

## Discussion

To feed the rapidly increasing global population, improving the wheat yield potential remains a major goal of breeding efforts (Reynolds et al., 2009). Manipulating the duration of both the preanthesis developmental phase and stem elongation is an important goal for developing improved wheat varieties with better adaptation and yield potential (Borràs-Gelonch et al., 2011; Guo et al., 2018). The duration of the stem elongation stage is considered a major factor that determines yield, as fertile florets are produced during this stage (Fischer, 2007). Several studies have suggested that extending the duration of the stem elongation phase would lead to an increase in the spike dry weight and the number of fertile florets at anthesis (Miralles et al., 2000; González et al., 2002; Borràs-Gelonch et al., 2011). Moreover, we did not identify negative effects on the seed number per spike in our *qd* mutant (data not shown), although this mutant presented a shortened stem elongation duration due to the accelerated stem development rate. A higher growth rate and higher yield could be synchronous in wheat (Xiao et al., 2018). In wheat breeding practices, an extended duration of the stem elongation phase easily results in late flowering and maturation. The *qd* mutant could be used to enrich the genetic capacity for the critical phase of stem elongation.

Wheat stem development is heavily determined by hormone signaling pathways, of which gibberellin (GA) signaling is the most important pathway for elite variety development and adaptation (Wu et al., 2021) (Richards et al., 2001). The Green Revolution genes *Rht-B1b* and *Rht-D1b* significantly contributed to improving the harvest index and seed production. The DELLA proteins encoded by the *Rht-B1b* and *Rht-D1b* genes reduced the wheat plant height by altering GA sensitivity (Peng et al., 1999). Translational reinitiation of the main open reading frame of these two genes was shown to block the GA signaling pathway, and this effect was determined to be dominant (Van De Velde et al., 2021). There is an *Rht-B1b* allele in WT plants and an *Rht-B1a* allele in *qd* mutants (Zhang et al., 2016). Moreover, in this study, the phenotype of the F_1_ plants (as shown in Figure 3) and some plants in the segregating population (as shown in Figure 6) with the *Rht-B1b* allele was the same as that of the *qd* mutant. These results indicated that the *qd1* gene functions upstream of the *Rht-B1* gene. On the other hand, the *Rht-B1b* and *Rht-D1b* genes have been shown to lead to an apparent trade-off between yield and nitrogen use efficiency in high-yielding varieties (Wu et al., 2021). In addition, these green revolution genes have some negative effects on coleoptile length (Li et al., 2011), anther size (Okada et al., 2019), and Fusarium head blight resistance (Srinivasachary et al., 2008). Whether the *qd1* gene that was finely mapped in this study could overcome the negative effects of the *Rht-B1b* and *Rht-D1b* genes remains unknown. The expression pattern of TraesCS4B02G042300 was concurrent with the phenotypes of *qd* and the WT (as shown in Figure 7). This gene was predicted to encode a putative oxysterol-binding protein (OSBP), which belongs to a family of sterol and phosphoinositide binding/transfer proteins in eukaryotes. The expression of OSBP is necessarily under hormone, light intensity, and temperature conditions in Arabidopsis (Umate, 2011). The mechanism of how this OSBP encoding gene regulated stem elongation through hormone or temperature responding pathway needs further exploration.

Previous increases in the yield potential of wheat have largely resulted from improvements in agronomic practices and harvest indexes. However, further increasing the harvest index is unlikely. A new opportunity to increase the yield potential involves increasing the production of biomass (Parry et al., 2010). Li *et al* showed that biomass can be increased by improving photosynthesis in maize (Li et al., 2020). In the present study, compared with the WT plants, *qd* mutants had a much greater fresh stem weight and dry matter weight at the booting stage, although there were no differences at the filling stage (Figure 2). The mechanism by which the *qd1* gene drives increased biomass production at certain times and the value of this role in breeding programs need to be further characterized. The *qd1* gene and associated molecular markers developed in this study could be used as targets for further improvement of stem growth traits in wheat.

## Data Availability

The datasets presented in this study can be found in online repositories. The names of the repository/repositories and accession number(s) can be found below: https://www.ncbi.nlm.nih.gov/, PRJNA745496.
