# Supplementary material for: Fine Mapping of qd1, a Dominant Gene that Regulates Stem Elongation in Bread Wheat
Source: Front Genet. 2021 Nov 29;12:793572. doi: 10.3389/fgene.2021.793572 (PMC8667865; doi:10.3389/fgene.2021.793572)
Supplement: Supplementary file 1 [file Table1.DOCX]

Supplementary table S1. Primers used for qPCR

Gene-ID Forward primer sequences (5'-3’) Reverse primer sequences (5'-3')

TraesCS4B02G042300 TTGCCAGGTCAGTCATCAT TGCTCTTCAGGTTGCTATCT

TraesCS4B02G042400 CTGTGGCTTACGCTTCAT TAGAGAGATTCCCGCATACT

TraesCS4B02G042500 GCCACCTCCTAATCAATCG CCAGCAACCCAACTATCG

TraesCS4B02G042600 CTTGAATTGGCTCTCCTACT ATTGGCACATACTCCTCTG

Actin GAGACTGTGAGGGACTACCAGGA AATACCAAGAATGCCAACACCAC
