# Supplementary material for: Fine Mapping of qd1, a Dominant Gene that Regulates Stem Elongation in Bread Wheat
Source: Front Genet. 2021 Nov 29;12:793572. doi: 10.3389/fgene.2021.793572 (PMC8667865; doi:10.3389/fgene.2021.793572)
Supplement: Supplementary file 2 [file Table2.DOCX]

Supplementary table S2. KASP primer sequences used in this study

| Primer name | SNP position | Forward primer sequences (5'-3') | Reverse primer sequences (5'-3') |
| --- | --- | --- | --- |
| ch4b1 | 13,284,383 | tacaacgtCaGggacttcctC[G/C] | tcgtcgctgggcacgaaG |
| ch4b2 | 18,011,897 | gccTGgctccTatgttttg[G/T] | atCgCaTtagttctTcCTgCT |
| ch4b3 | 39,016,344 | tcAacaaacttttgcggaTg[C/T] | aatgaacaactCaaaagcaaagatC |
| ch4b4 | 55,323,090 | CTGGATCTTGCGAACAATTC[G/C] | TGGATCGTGTGGGAGGCT |
| ch4b5 | 122,301,117 | ggggatgtttgtgcaacc[G/A] | gagttcatgtccaccaccaC |
| ch4b6 | 171,815,747 | gatttgtacgtGctattatgAgtg[C/T] | tgtaagaagagtaCgatacaGgcA |
| ch4b7 | 340,640,832 | tgggtttgcaatatgcctct[G/C] | cattgagcagattttagcatactcG |
| ch4b8 | 428,009,260 | tgttaccagctttctacatgga[T/C] | accatctagccaatcagtgatC |
| ch4b9 | 480,593,848 | ttcctcGtctcaTgtttctgt[C/T] | tgccgctaaatagaagggagA |
| *Rht-B1* | 30,861,571 | cccatggccatctccagct[G/A] | tcgggtacaaggtgcgggcg |
| bsph1 | 14,014,497 | ACAATGGATGTTTCTTGCACCT[A/T] | TCAACTTAACCTGTGCCTCTT |
| bsph2 | 17,367,184 | CGCTTGAATTTTGCTAGTTTGAG[A/T] | AGTTAACAGACATGAAGAAGCTTC |
| bsph3 | 20,720,959 | TTGTTAGTAACCCAATAACACGGT[C/T] | GCCCTTGCTTAAACTGGGTC |
| bsph4 | 21,380,766 | CCACCTGAGAGTAATGAGTTGGTA[A/T] | CAGTGGGAGTAAGAGATAGTACTAC |
| bsph5 | 26,797,417 | GCAATATATTGATTTGTGTTGGCC[G/A] | CACTCTGCTGTAAGCTGGG |
| bsph6 | 31,884,565 | TGAGGCGAAGGAGAGGAG[A/G] | AGCTGAGGATGATCCATTCG |
| bsph7 | 37,696,213 | GGAGGAGGAATTCACCGC[A/C] | CCACACAAGCATAAGGTGACA |
| bsph8 | 41,013,810 | TGCTGATGATATAGCTGCTG[C/G] | CCAGAACAAGAGAAGTAGCAGT |
| bsph9 | 44,185,162 | ACTTGATGCACTGATACCAAGT[C/T] | CATCTGCATCATGTTCATACGAC |
| bsph10 | 68,405,651 | GGTTGTGGGTGGATGGATAAA[G/A] | GAGGCAGACATCGCCCAC |
| bsph11 | 78,025,851 | CCTGGCAGAAGACGAAGG[T/C] | TTTGGGCTTGAAGGCTCCA |
| bsph12 | 87,413,474 | CTCTGCACCGGAAGAAAC[C/T] | GGTCCTTGGAGGTCTGCTC |
| bsph13 | 115,100,484 | CACCGGGACTTGAGCTCT[A/G] | CGTGCTTCTTCATCCAGCAG |
| bsph14 | 132,294,376 | TGTGCTGGAGATCCTCTG[C/G] | GATGATGACCCTCTCAAGGC |
| wgph1 | 26,826,017 | tgcagacaagtaaggcAttG [A/G] | GctggcatgcatAatCagTG |
| wgph2 | 28,860,971 | tccAGcaaggggaGagGa [G/A] | CcGtcgtgctTCCGAagA |
| wgph3 | 28,950,908 | aataaaacagcagtggcagaa [G/A] | gccGctgttgttttattgagttT |
| wgph4 | 28,951,344 | ggagaacaggCgAaaGtaatg [C/A] | atgcagcaccaattctattGaG |
| wgph5 | 28,952,928 | GgAaaagggagagggaAaagat [C/A] | acttgtaCttcCgttagcaacaT |
| wgph6 | 28,954,174 | cctctacggatggaaggag[C/T] | GgataatctgcttcataggtctcT |
| wgph7 | 28,954,337 | cCtagtccactgttCattctc [G/C] | ggTgaagtttaaggtgttaCgG |
| wgph8 | 28,954,472 | cTccatgtagtcgttacttCC [A/G] | ggcTatttgGtgattcaaTaGgtaA |
| wgph9 | 28,954,548 | gataCtCgaggccCctaca [G/T] | caacaaccattggatgatgactG |
| wgph10 | 28,955,067 | tgacaaacaaactgcAaagttaC [G/A] | tgatcgcaaTcctcatcaggtatA |
| wgph11 | 28,955,900 | accaTTcattgatcatttggtAg [C/T] | tCACaTTatTcAtTcTcCaCCtGaT |
| wgph12 | 28,956,015 | acatgacaaattaattgcttcagc [T/C] | caAAgacGacCtataactggtcA |
| wgph13 | 28,956,741 | aagcatgtgaatcgAccaGa [G/A] | acttttggattggctgCattaG |
| wgph14 | 28,956,825 | tgtgtcaaacatgcttgtaaG [C/T] | cGTcaggccatggttcaA |
| wgph15 | 28,959,985 | gaaaatgtcAgggcagccT[T/C] | ggagagtgggtggaaaccTA |
| wgph16 | 28,960,494 | tgTtttgaaggtgaatgttgtttt [G/C] | TTgcttagCagTTaCtAGCActcG |
| wgph17 | 29,152,559 | tgaatgcgagatCtTAGagtaac [C/A] | acgagcgcccgtgTataG |
|  |  |  |  |
| wgph18 | 29,152,570 | acGtgTgcGgttactCTAa [G/A] | caAaaggGctagaCtaAacgC |
| wgph19 | 29,267,275 | cCtggagaatgtcgattatccc [G/A] | cgaGgtgcaCgacgtgatC |
| wgph20 | 29,427,019 | ctAgtcgccttggtccca [C/A] | gaatccGctcaAactaCtccatA |
| wgph21 | 29,499,525 | ctcGagaCtacacacAcaCa[T/C] | ctagcatgtgtaggttgcaatT |
| wgph22 | 30,189,870 | gctGtctgcTtgtgctttattat [C/T] | catattacatccactgggcatcT |
| wgph23 | 30,964,789 | aGtcGtccaTggGTgag [A/G] | CggaacaGcccccaagcA |
| wgph24 | 31,080,470 | tgcacatttCgGtgagaCc [G/A] | cTgagatggCCttgcaaAcA |
| wgph25 | 31,883,804 | ccactaCGtgcatccttaca [C/T] | gcaAaTCataGAcaaaagcgcA |
